# Leadership Experiences of Nurse Managers in a Saudi Ministry of Health Hospital: A Focused Ethnographic Study

**DOI:** 10.1155/jonm/8033761

**Published:** 2025-12-03

**Authors:** Ibrahim Naif Alenezi

**Affiliations:** ^1^ Public Health Nursing Department, College of Nursing, Northern Border University, Arar, Saudi Arabia, nbu.edu.sa

**Keywords:** ethnography, framework analysis, leadership, management, nurse managers, nursing, Vision 2030, wasta

## Abstract

**Background:**

One of the key priorities of Saudi Vision 2030 is to ensure that all Saudi citizens have access to high‐quality healthcare, but this goal is constrained by international and domestic nursing staff shortages. Nursing directors have recommended introducing a professional career path focused on developing nurses’ leadership skills. Despite recognition of nurse managers’ key role in recruiting, retaining, and engaging nurses, few studies have explored how Saudi managers acquire and deploy leadership skills. This study explores how nurse managers view and experience workplace leadership.

**Methods:**

This ethnographic study examined nurse managers’ perceptions and experiences of leadership development in a Saudi hospital affiliated with the Ministry of Health. Qualitative data were collected during periodic visits over eight months, including workplace and continuing medical education observations, document examinations, and informal and semistructured interviews with 21 nurse managers. A framework analysis approach was used to interpret and summarize the data.

**Findings:**

The data were organized into four major themes: imbalanced power dynamics, no shared vision, workplace rituals and behaviors, and the need for learning. The findings indicated a power imbalance, consistent with international studies. The hospital had hierarchical and transactional management structures and a culturally pervasive leadership approach framed by *wasta* (“middleman” or “go‐between” in Arabic)*.* Such nepotism, normalized in Saudi business practice, is officially recognized and condemned as corrupt by state legislators. The Saudi government is rigorously combating this practice through strict regulations and enforcement. A climate encouraging adherence with the directorate’s guidelines led managers to comply, even though they employed strategies to subvert some of its authority.

**Conclusion:**

Wasta was used to enhance performance, obtain rewards, secure favorable work assignments, gain meritless promotions, and allocate staff arbitrarily. Paradoxically, while managers criticized the capricious use of wasta, they also employed it to secure what they believed they deserved, sometimes at the expense of their colleagues’ careers.

## 1. Introduction

Although the Kingdom of Saudi Arabia (KSA) is one of the world’s most successful economies and allocates significant resources to healthcare, it faces major structural challenges. This stems from a rapid increase in demand for health services due to rising fertility rates, a growing population, increasing lifestyle diseases, and longer life expectancy [1–3]. A key objective of Saudi Vision for the Future 2030 (2016) is to enhance healthcare and individual well‐being for all citizens. However, reliance on an expatriate workforce and a high nurse turnover rate impede meeting this objective [4–6].

In response to ongoing nurse shortages, the Saudi government has developed a national workforce strategy to enhance professional education and ensure healthcare providers meet international standards [7]. Despite significant advancements in nursing education and clinical practice, Al‐Dossary [8] noted that major workforce and leadership challenges persist. KSA’s policy to recruit, employ, and retain more Saudi nurses [9, 10] has not curbed the high rates of nurse turnover [6, 11].

Although Saudi‐only nursing management jobs have expanded, clinical nursing shortages persist, compounded by challenges in pre‐ and postqualification education to meet workforce demand. Aldossary [12] and Alshammari [13] attributed difficulties in attracting and retaining Saudi nurses to an unclear scope of nursing practice and a lack of leadership, which impede professional growth and hinder the development of the nursing profession.

In the modern era, nurse managers must lead effectively to support their staff and create a healthy work environment and stable workforce. Saudi managers are no exception [6, 14]. Numerous studies confirm that the most significant determinants of nurse dissatisfaction in KSA, leading to intentions to leave and turnover, are associated with service management and leadership [15–19].

Saudi nursing leaders must build positive relationships with staff nurses to enhance organizational commitment, job satisfaction, staff retention, and patient safety [16, 18, 20]. Al‐Yami et al. [20] found that work commitment and job satisfaction improve when nurses work with transformational leaders who inspire and motivate staff, aligning individual goals with organizational objectives [21]. Al‐Yami et al.’s finding [20] aligns with Abualrub and Alghamdi [15], who concluded that greater awareness of factors ensuring nurse satisfaction would lead Saudi leaders to implement changes that enhance nurse and patient satisfaction, improving nurse retention.

As KSA has a substantial multicultural nursing workforce, managers must be well‐educated and culturally sensitive leaders for future strategic roles [19, 22]. Current nursing leaders are calling for meaningful changes and independent roles [23]. Tumulty [24] urged the Ministry of Health (MOH) to ensure proper representation of nursing representatives in its strategic hierarchy and decision‐making processes, as well as in national leadership development programs. Unlike other countries, there has been no national rollout of leadership development programs for nurse managers. Currently, only local hospital–based in‐service continuing medical education (CME) programs exist, which may not include leadership skills and lack quality assurance [25, 26]. Leadership skills cannot be developed through a single training event or in a fixed timeframe; rather, they require continuous education and ongoing practice opportunities [20]. As Alluhidan et al. [23] argued, the country’s ambitions for high‐quality healthcare depend on recognizing the contribution of developing nurses’ leadership capability. However, a lack of reliable data on nursing leadership may limit Saudi nurses’ ability to compete with international counterparts in professional advancement [27]. Addressing this issue could significantly enhance workforce development and patient outcomes.

International research provides insights into leadership and organizational culture. A UK case examined transformational leadership and management approach in a dental practice, demonstrating how organizational settings influence leadership styles [28]. Similarly, a Finnish study identified ineffective physician leaders, highlighting how nepotism and poor leadership can undermine healthcare delivery [29]. Together, these studies place the current inquiry within broader international discussions on leadership and organizational culture.

This lack of structured development reflects the complexity of defining and studying leadership, warranting a conceptual framework. Social constructionist notions have emerged in response to the conceptual complexities of disparate approaches to leadership [30–36]. Social constructionists situate leadership in the observer’s mind rather than in the leader’s personal qualities [32], which may explain the absence of a standard definition. As a social construct, leadership is understood as the meaning created by dialog within groups in a particular context [37]. Fairhurst and Grant [38] noted that many social constructionists view language as the process through which meaning is negotiated and formed. The basic assumption is that leadership is a socially constructed notion, co‐constructed [39] between those who sometimes lead and those who follow [28].

Focusing on leadership, Grint [40] stated that leaders establish a reality that transforms decisions into actions. Uhl‐Bien et al. [41] suggested that followers must be open to influence by a phenomenon rather than by individuals. Therefore, nurse managers’ leadership experiences in the workplace are not embodied by any one leader but result from collective social discourse and interactions over time. The hospital community is shaped by how nurse managers develop their understanding and make sense of their roles.

Given these contextual and conceptual factors, this study aimed to explore nurse managers’ perceptions and experiences of leadership in the workplace, focusing on the social and organizational influences shaping these views.

## 2. Materials and Methods

### 2.1. Research Design

Since little is known about how Saudi nurse managers develop their leadership skills within the hospital context, this study conducted a focused ethnography (FE) to capture the experiences of these managers. Through this, the study aims to help the MOH and hospital decision‐makers understand nurse managers’ needs and aspirations for leadership development. It also evaluates the facilitators and barriers to leadership development faced by managers and outlines necessary changes for developing Saudi nurse leaders. Specifically, the aim was to explore nurse managers’ perceptions and experiences regarding leadership development in a MOH‐affiliated hospital in KSA through FE data collection methods.

First described by the German sociologist Knoblauch [42], FE is an efficient approach to studying highly differentiated cultural groups. As Cruz and Higginbottom [43] explained, it narrows a study’s focus to a single cultural trait instead of a comprehensive cultural analysis. This shorter time frame allowed for a focus on nursing leadership in the current study, rather than examining all the elements of nursing management or practice. The FE approach was suitable for several reasons. First, it enabled the researcher to concentrate on nurse managers in a regional hospital in Saudi Arabia. Second, as a staff nurse and Saudi national, the researcher was already immersed in the specific culture being studied. An understanding of leadership in nursing management requires sensitive, immersive, and in‐depth methods for thorough investigation [43].

Field visits (8 months and 17 days) were made to collect data through participant observations at management meetings, clinical departments, and CME leadership lectures. Semistructured interviews were conducted with 21 nurse managers in various roles, and an analysis of relevant hospital documentation was performed. Through this, the study achieved immersion in the nursing management culture of a MOH hospital in northern Saudi Arabia.

The study approach emphasized communicative experiences and activities over the social aspects of traditional ethnography [42–44]. The culture of Saudi Arabia focuses on social organization, while the observed group consists of qualified nurse managers at various managerial levels. This study’s research involved interpreting participants’ social interactions, behaviors, and perceptions regarding their leadership development experiences in a hospital. In addition to leadership dynamics, this study captured events and processes within the hospital related to leadership practices and explored whether the environment facilitated leadership. The aim was to demonstrate the complexity of leadership and the impacts of health policy, local culture, organizational political structure, and other demographic variables [45–47].

Finally, the FE approach effectively answers specific, detailed questions, characterized by the “conceptual orientation of a single researcher” [48, p.3]. It is a problem‐specific method focused on a discrete organization or social group, involving episodic participant observation and a small group of selected participants with in‐depth knowledge [42, 48].

### 2.2. Study Setting

Like all public hospitals in Saudi Arabia, the hospital under study is affiliated with the MOH, which supervises primary healthcare clinics and private hospitals. The MOH formulates health policies, develops strategic plans, and hires healthcare personnel [47–51]. Nurses must hold a license to practice and complete CME credit hours to maintain their licensure with the registering body [53].

While Arabic is the official language of Saudi Arabia, most nurses speak English as a second language [54] and must communicate with a multicultural workforce. However, most patients do not speak English, which can hinder communication between healthcare workers from other parts of the world and patients [55].

With the exception of the nursing director, the hospital’s top administration consists of male directors and deputies. Clinical and CME department managers are predominantly female, holding nonclinical roles such as charge nurses, head nurses, and supervisors [24].

During the first phase of fieldwork, the researcher immersed himself in the nursing departments to gain first‐hand experience of the professional structure and social duties of nurse managers while recording reflexive memos. Gender separation, pervasive in Saudi culture, was evident throughout the hospital, as both male and female Saudi nurses and their managers adhered to this norm. Despite an overall shift and some flexibility in healthcare settings, several authors have found that gender separation persists, particularly among female Saudi nurses [10, 16, 56]. Although male and female nurses maintained physical distance, they regularly communicated through a WhatsApp group to coordinate their work.

### 2.3. Data Collection

A range of qualitative methods was used to collect data for three reasons: (1) to avoid bias and limitations associated with a single‐method approach, (2) to ensure reliable and credible data, and (3) to build a comprehensive picture of leadership in the studied environment. The triangulation of data collection facilitated the discovery of fundamental phenomena and ensured the validity of the findings [57, 58]. Data were gathered from several sources, including ward rounds as a participant–observer, training events, informal and formal semistructured interviews, field notes, reflexive memos, and the analysis of policy documents.

Interviews began with a summary of the research, ensuring that participation was voluntary. They were informed that the interviews would be audio recorded, de‐identified, and that they could withdraw at any point. The interviews followed a guide developed based on the study’s aim and early observation field notes. All semistructured interviews included questions such as, “Can you tell me about your current position?,” “What is your role in your current position?,” “How would you define leadership?,” and “Can you describe the team you manage?”

The nurse managers participated in five informal interviews and one‐to‐one interviews. Individual nurse managers were selected for one‐to‐one interviews using purposive sampling, with the aim of including participants with relevant nursing leadership experience, specifically those who had completed a hospital leadership development program. This approach ensured the selection of information‐rich cases that provided in‐depth insights, aligning with the qualitative research design [58]. The small sample size (*n* = 21) yielded rich data [42, 59] and achieved the data saturation needed for reliable findings [60].

### 2.4. Reflexivity

The researcher’s insider position within the Saudi nursing context facilitated access and rapport but also posed a risk of bias. To manage this, a reflexive stance was maintained throughout the study. A detailed journal recorded assumptions, emotional responses, and methodological decisions, with entries revisited to identify and challenge preconceptions, ensuring that the interpretations remained tied to participants’ accounts. Credibility was strengthened through triangulation of multiple data sources and peer debriefing, which introduced alternative viewpoints. During data collection, the researcher navigated an insider–outsider continuum, using cultural knowledge as needed while remaining alert to emergent insights. This ongoing reflexive practice ensured that the researcher’s professional background informed the study’s findings without unduly shaping them.

### 2.5. Fieldwork

The researcher conducted intermittent field visits over 8 months and 17 days, totaling approximately 45.5 h of direct observation. This included 65 departmental visits (40 h, 26 min), four formal meetings (2 h, 10 min), and five leadership development events (2 h, 56 min). Formal semistructured interviews were conducted with 21 nurse managers, each averaging 76 min. This engagement provided opportunities to observe managerial practices and organizational dynamics, enhancing the depth, credibility, and cultural immersion essential for focused ethnographic research and enriching the understanding of the phenomenon studied.

### 2.6. Data Analysis

The information collected during interviews was triangulated with observations of various events and documentary analysis, producing comprehensive data for analysis [61]. Each data collection method facilitated the collation and comparison of information about leadership events, yielding a well‐rounded understanding of the subject. Problems identified during a workshop could be examined in detail during a semistructured interview or developed further by referring to documents. This does not imply that one data collection method is superior; rather, each requires independent analysis and comparison. Consequently, the convergence of the findings confirms their validity, despite their differences [62–64].

For data analysis, framework analysis (FA) was chosen because of its suitability for analyzing data linked to an interpretive methodology [67]. Although not closely associated with any particular methodology [66], FA is frequently used in nursing research studies [67].

FA provides a structure for coding data before examining each category. This overview can undergo detailed evaluation to identify underlying elements through careful inductive analysis [66]. Researchers using FA must continually question their initial data and navigate between different levels, following the spiral configuration of the data abstraction process. This approach enables recognition of similarities and differences, facilitating the confirmation of meaning and importance in addressing research questions [67]. Ritchie and Lewis [68] noted that a key strength of FA is its comprehensive yet flexible approach, which is systematic and highlights the significance of links between the data.

## 3. Results

**Figure 1:**
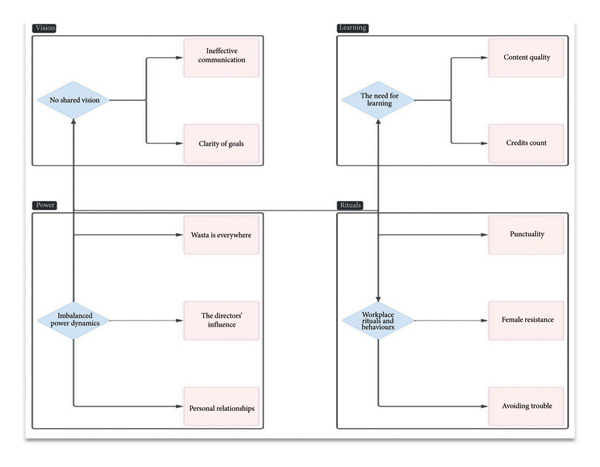
Major themes and subthemes.

The data were organized into four major themes: imbalanced power dynamics, no shared vision, workplace rituals and behaviors, and need for learning (Figure 1). The first major theme, comprising three subthemes, Wasta is everywhere, personal relationships, and directors’ influence, will be discussed in this study.

### 3.1. Imbalanced Power Dynamics

The hospital organization was a highly centralized structure, where a few directors held all the power and made decisions, and employees were expected to follow their guidance. Using Hofstede et al.’s [69] power‐distance analogy, the director’s interpersonal influence on nurse managers depended on their acceptance of this disproportionate power. This power can be granted to certain individuals, leading to an imbalance.

#### 3.1.1. Subtheme 1: Wasta Is Everywhere

The concept of wasta, meaning “middleman” or “go‐between” in Arabic, refers to a form of indigenous nepotism. Findings related to this concept illustrate the power imbalance within the hospital.

During a meeting, a senior manager said: “(smiling) we should put individuals with a strong back in a good position, so they will have our back” (Department 16 observation). This was an overt declaration that employing someone as a favor comes with the expectation of receiving favors in return.

When wasta was mentioned during the interviews with managers, it was nearly always accompanied by nervous laughter or other nonverbal gestures. For example,

As I told you, if you have wasta or certain years of experience, with a little wasta of course (giggling), then you will be more likely to get promoted, not only in nursing but also in other hospital departments. Wasta is stronger than anything else. I believe that wasta is not “good practice,” but if I don’t use it, somebody who doesn’t deserve the position might take it (raising eyebrows, widely opening arms). (P2)

This culturally pervasive power was normalized by a form of capricious leadership attributed to wasta*.* Regarded as a form of Arab nepotism, wasta is normalized in Saudi business practice, despite now being officially recognized and condemned as corrupt by state legislators. Most hospital nurse managers were ambivalent about using it widely.

#### 3.1.2. Subtheme 2: Personal Relationships

The power of personal relationships often determines who receives opportunities, resources, or benefits. Foucault [70] argued that when power is exercised, it becomes exposed, allowing it to be resisted. This means nurses can leverage this exposure, regardless of their position in the hospital. They accept this exposure or use it to their advantage, confront and condemn it, or silently communicate passive resistance. For example, in one observation, managers accepted that change was a problem but emphasized the power inherent in their relationships as

…don’t pretend that you don’t know that … she has some relatives in the HR dept. So, even if I give her feedback, what’s the point? (Looking dissatisfied). Nothing will happen; everything will stay as it is, and this is our problem. (P22)

During interviews, it was again accepted and used as an advantage as

I know personally, and please, this is top‐secret (laughing); I know some people here get promoted and receive incentives because they know someone up there (moves their right hand towards the ceiling). This is unfair, isn’t it, but things will be better in the future (P5)

These data show that personal relationships hold significant power in social and professional settings and may be viewed as an abuse of power by Western standards. However, they can also serve as a means of arbitration or mediation to obtain hard‐to‐secure benefits. In the Middle East, expediting official paperwork and procedures is sometimes deemed necessary.

#### 3.1.3. Subtheme 3: Directors’ Influence

This subtheme is distinct, as it pertains to the perceived influence of wasta power exerted by external and hospital executives. During an interview, a manager drew the hospital’s organizational chart, a hierarchical structure with the hospital director at the top and two deputies beneath. The nursing director reported to one of the deputies in a middle management position.

Assuming that this chart demonstrated the accepted line of management within the hospital, if not the leadership accountability and responsibility, managers were asked during the interviews if that meant anyone wanting to contact the hospital director had to do so through the deputy or nursing director. The answer was surprising:

Oh no, this is just the chart (laughing loudly, shaking head). Please, I will explain it to you. You are my guest today. This chart or hierarchy, if you like, has existed in all hospitals throughout the Kingdom, of course, I mean hospitals of the Ministry of Health, because the Ministry created this hierarchy. (P10)

Interestingly, external power could be imposed on the hospital director and have the same effect as on the directors’ staff. For example, one manager explained:

When someone even bigger than the hospital director, let’s say from the general directorate, wants to transfer someone, the hospital director, from my viewpoint, will let it go, overlook it. (P4)

As demonstrated in these data, and in line with the literature discussed in the introduction to this study, managerial power can be socially constructed. This was apparent in observations where managers reported that the hospital executive influenced their authority or when they shared how this impacted their morale and motivation:

…there is no need for me to play the role of nurse manager because, yes, my title is nurse manager, but in fact, I most of the time do what my superiors order me. I am sorry you may not want to hear this … (P10)

During a field observation, a manager was observed flaunting their wasta by bypassing other line managers and dealing directly with the hospital director. When asked how they managed this, they replied, “…thank God, I have a good relationship with the hospital director and he believes in me” (Observation Department 3).

## 4. Discussion

The study findings indicate that leadership practice in the hospital is shaped by the interplay of national and organizational culture, relational obligations (notably wasta), and power dynamics. Nurse managers navigated expectations of reciprocity and in‐group affiliation while engaging with the directorate’s authority, leading to varied experiences, such as enhanced access to resources for some and perceptions of differential treatment for others. While wasta was prevalent in interactions, it was also contested, reflecting ambivalence that complicates efforts to promote transparent, merit‐oriented leadership. In the national policy context, practices associated with wasta (such as intercession or misuse of influence) are criminalized as corrupt under Saudi Arabia’s antibribery law [71].

Saudi health services currently operate within a framework of demographic diversification and increased international engagement, characterized by a diverse nursing workforce and reliance on foreign personnel [3, 24]. This raises the need for transparent, audit‐ready, and fair practices, with a lower tolerance for bias or informal influence. The study focuses on power imbalance and informal gatekeeping, which affect trust, employee retention, and organizational learning, potentially conflicting with the governance goals of Saudi Vision 2030.

The hospital conformed to the description of an Arab organization, as defined by Hofstede [69], who classified organizations into four dimensions based on the social structure of management culture. Culture is a form of: “…collective programming of the mind that distinguishes the members of one group or category of people from others” [72], (p.6).

This programming influences mental models or thinking patterns expressed as fear, anger, love, joy, sadness, and shame. These models are culturally learned, observed, and modified from social environments and personal experiences. Obeidat et al. [73] noted that, despite criticism, these concepts remain relevant. Although national and organizational cultures may share similarities, they differ; various factors influence why people join, stay in, or leave these cultures. However, no one is fully immersed in them forever.

Offering support by employing someone as a favor often carries the expectation of reciprocal favors. Tlaiss and Kauser [74] stated that doing favors for influential individuals can significantly enhance a manager’s connections and access to benefits for themselves and their friends or family [74–76].

Wasta was reported to enhance financial rewards, influence favorable work assignments, and lead to meritless promotions and dubious staffing allocations. It was not an innocuous concept. Nurse managers used wasta to maintain in‐group relationships with the directorate, often at the expense of out‐group colleagues [77]. This finding parallels Algumzi’s [78] observation that wasta was frequently experienced and personally utilized to obtain what individuals felt they deserved. While all managers disparaged wasta, those less connected voiced their disapproval more vehemently.

The paradox of wasta being widely used yet disparaged is common, as is the denial of power abuse [78]. In 2015, the National Anticorruption Commission (Nazaha) identified wasta as the most corrupt practice in Saudi society, attributing its prevalence to the country’s administrative complexity and outdated laws [79].

However, Weir et al. [80] argued that wasta is a lifestyle choice in the country, where favoritism occurs based on factors such as race, region, religion, tribe, or family. Similar practices have been observed in Europe, Africa, and China [75]. In Saudi society, wasta is prioritized over organizational goals and is deeply embedded in relational infrastructures. It is not an Islamic tradition and should not be assessed from a religious or moral perspective; judgments should focus on its cultural foundations. Essentially, wasta resembles a form of Western social capital, with its use for good or ill depending on individuals’ intentions [81, 82].

Nurse managers’ compliance, collusion, or resistance to the directorate’s status and authority were often influenced by their interpersonal relationships. These relationships are often influenced by Arabic cultural factors, such as tribe and extended kinship [19, 73, 83, 84].

In Subtheme 3, it was found that Foucault’s [70] discourse on power had been altered to enable managers to undermine the official power structure. This occurred through micropractices, such as selective reading of policy, controlling information flows, and mobilizing informal networks, which redirected decisions while maintaining a facade of procedural compliance. Consequently, formal hierarchy and actual influence diverged, complicating accountability and making leadership outcomes dependent on access and visibility rather than formal authority. This may require more precise criteria for routine decisions, transparent decision trails, and established escalation pathways to align informal influence with formal responsibility [84].

### 4.1. Recommendations for Policy and Practice

The findings of this study have significant implications for Saudi Vision 2030, which emphasizes transparent governance, workforce development, and adherence to reputable international standards [2, 8]. To realize these priorities, organizations should establish clear and robust governance systems that minimize reliance on informal influence and strengthen accountability. Collectively, these measures also constitute a pathway for leadership development, cultivating competencies in ethical and inclusive leadership, accountability, conflict management, and fostering psychological safety.

To address wasta‐related practices, which Nazaha (Oversight and Anticorruption Authority) designates as corruption [85], transparent governance should incorporate confidential reporting channels with protection from retaliation, published and merit‐based criteria for recruitment, allocation, appraisal and promotion, auditable decision records, conflict‐of‐interest disclosures, and periodic independent reviews of high‐stakes personnel decisions. These controls make informal influence difficult to maintain and provide a justifiable decision record.

Comparable mechanisms are codified internationally. In the United Kingdom’s National Health Service (NHS), equality, diversity, and human rights are included as part of the statutory and mandatory curriculum outlined by the core skills training framework (CSTF) and are delivered through NHS e‐Learning for Health (e‐LfH), with modules that explicitly satisfy statutory and mandatory training requirements [86, 87]. In addition, the Violence Prevention and Reduction Standard establishes organizationwide expectations for leadership, governance and assurance, workforce training, data collection, interventions, and evaluation to foster psychologically safe workplaces and conflict resolution practices [88]. In Australia, the National Safety and Quality Health Service (NSQHS) Standards, notably the Clinical Governance Standard, call for integrated leadership, transparent governance processes, routine assurance activities, and corrective action frameworks that directly support fair, auditable decisions and psychological safety [89]. In Canada, federal obligations under the Directive on the Prevention and Resolution of Workplace Harassment and Violence and the Work Place Harassment and Violence Prevention Regulations (SOR/2020‐130) mandate prevention training, confidential reporting channels, documented investigation procedures, and remedies; the Employment Equity Act further establishes an equity‐ and merit‐based framework for recruitment and promotion across federally regulated employers [90–92].

Operationally, organizations should routinely monitor key indicators, such as grievance and complaint trends, appeal outcomes, the composition and rotation of selection panels, as well as intent‐to‐leave and turnover metrics, and utilize these data to trigger corrective actions and foster continuous improvement. Coordination with regulators and professional bodies helps harmonize definitions, guidance, and enforcement. These measures collectively strengthen procedural fairness, promote psychological safety, and link everyday management practices to the transformational goals of Vision 2030. Such measures align with Vision 2030 by emphasizing transparent governance, workforce development, and adherence to credible international standards [2, 8]. This entails clear criteria, consistent application, and transparent decision trails, making dependence on informal influence less feasible. Mandatory training in equality, diversity, inclusion, and conflict resolution, together with accredited leadership development and ongoing professional growth, would enhance procedural fairness and psychological safety. Routine monitoring of signals, such as complaints concerning influence, appeal outcomes, panel composition, intent to leave, and turnover, should prompt corrective measures and support organizational learning. Collaborative efforts with regulators and professional organizations help unify definitions, guidance, and enforcement. Collectively, these steps make informal influence harder to maintain and connect daily management practices with the objectives of Saudi Vision 2030.

### 4.2. Strengths and Limitations of the Study

A key strength of this study was its multimethod qualitative examination of nurse managers’ leadership experiences. This approach aligns with the healthcare component of Saudi Vision 2030. The study’s broad scope and unique findings are consistent with the wider literature on national and international leadership.

However, the study has several limitations. Managers at MOH‐affiliated hospitals do not provide direct patient care. While focusing on managerial leadership within nursing, some clinical impacts of nurse managers’ leadership were included but not explored in depth, indicating a need for further studies. In addition, examining only one MOH‐affiliated hospital limits the generalizability of the findings to other Saudi hospitals not affiliated with the MOH, such as those associated with the armed forces, national guard, and the private sector.

## 5. Conclusion

This study provides a unique insight into how Saudi nurse managers negotiated leadership amidst formal hierarchies and informal influence, particularly the culturally pervasive yet contested practice of wasta. Everyday micropractices and relationships redirected decisions, impacting access to opportunities and perceptions of fairness. This influenced role clarity, morale, and ward climate. The findings suggest strengthening transparent, criteria‐based processes supported by clear audit trails and escalation pathways [93]. This alignment of informal influence with formal responsibility can foster merit‐oriented leadership. Addressing these relational dynamics is essential for effective leadership practices and achieving the healthcare system’s quality goals.

**Nomenclature**
KSA: Kingdom of Saudi Arabia
MOH: Ministry of Health
FE: Focused ethnography
FA: Framework analysis
CME: Continuous medical education
HMC: Hospital Management Committee

## Ethics Statement

Ethical approval for this study was obtained from the Ethics Committee of Research Panel (NURS037) and the Hospital Management Committee (HMC). Extended access to nurse manager participants was granted, and all methods adhered to relevant guidelines and regulations (e.g., Declaration of Helsinki). All observations and one‐to‐one interviews with nurse managers were preceded by a summary of the study’s aims and objectives, along with an explanation of the research process. This information was provided via a participant information sheet. Written consent forms were obtained before each interview and observation session.

## Consent

Please see the Ethics Statement.

## Disclosure

This manuscript has been previously presented as a preprint on Research Square [94].

## Conflicts of Interest

The author declares no conflicts of interest.

## Author Contributions

Ibrahim Naif Alenezi conceptualized the study’s aim, developed the design, conducted the fieldwork that consisted of multiple field visits, collected and analyzed the data, and prepared this manuscript for publication.

## Funding

This study was funded by the Deanship of Scientific Research at Northern Border University in Arar, Kingdom of Saudi Arabia (KSA), under project number “NBU‐FPEJ‐2025‐190‐01.”

## Data Availability

The author used primary data collected through semistructured interviews, informal discussions, and field notes. The data are in the author’s possession and are available upon legitimate request.
